# The controlled synthesis and DFT investigation of novel (0D)–(3D) ZnS/SiO_2_ heterostructures for photocatalytic applications†

**DOI:** 10.1039/d1ra02284a

**Published:** 2021-06-24

**Authors:** Mohamed F. Sanad, Ahmed Esmail Shalan, M. A. Ahmed, M. F. Abdel Messih

**Affiliations:** Chemistry Department, Faculty of Science, Ain Shams University Egypt mfsanad@miners.utep.edu; Department of Chemistry and Department of Environmental Sciences and Engineering, University of Texas at El Paso 500 West University Avenue El Paso Texas 79968 USA; Central Metallurgical Research and Development Institute (CMRDI) P.O. Box 87, Helwan Cairo 11422 Egypt a.shalan133@gmail.com ahmed.shalan@bcmaterials.net; BCMaterials, Basque Center for Materials, Applications and Nanostructures, Martina Casiano, UPV/EHU Science Park Barrio Sarriena s/n Leioa 48940 Spain

## Abstract

A ZnS/SiO_2_ photocatalyst was synthesized using a low-cost sol–gel wet chemical procedure. The as-synthesized ZnS/SiO_2_ nanocomposites with different molar ratios exhibited superior performance in the photodegradation of two organic dyes under UV irradiation, with complete degradation of both dyes after 2 hours of exposure to UV irradiation. The photocatalyst structure, microstructure, and surface area were studied using X-ray diffraction (XRD), high-resolution transmission electron microscopy (HRTEM), field emission scanning electron microscopy (FESEM), and nitrogen adsorption (*S*_BET_) studies. The results demonstrate that the ZnS/SiO_2_ photocatalyst with 15% ZnS content has a bandgap energy similar to that of ZnS alone with a higher surface area of approximately 150 m^2^ g^−1^, which effectively increases the number of active sites and improves the photocatalytic activity of the prepared material. The measured bandgap energies were compared with the theoretical values obtained using the density functional theory (DFT) method, and the values were found to be very similar, with a low error percentage. In the case of a high ZnS content (greater than 15%), active site blocking occurred, and the removal rate dropped below 50%. The obtained results indicate that the photocatalytic data are in good agreement with the experimental characterization results for the prepared materials, including the BET and XRD results, confirming a close association between the photocatalytic activity and the surface area of the fabricated photocatalyst.

## Abstract 2

A ZnS/SiO_2_ photocatalyst was synthesized with different molar ratios using a low-cost sol–gel wet chemical procedure exhibited superior performance in the photodegradation of organic dyes under UV irradiation.

## Graphical abstract

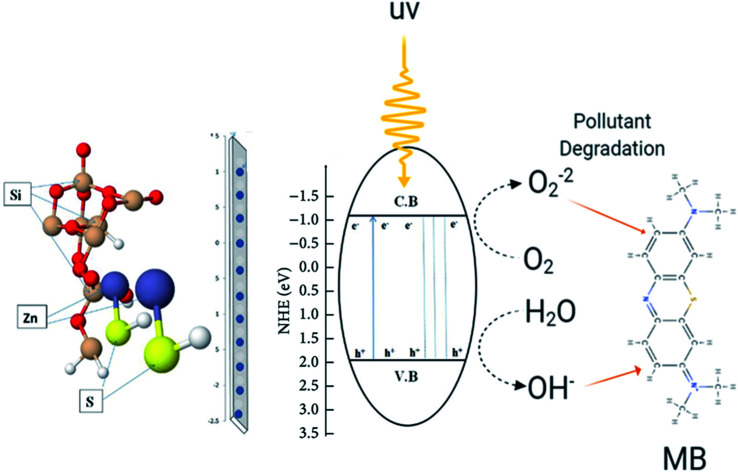

## 1. Introduction

The need for clean water is growing in all regions of the world, while renewable water resources have become limited due to the effects of climate change and human usage.^1–6^ This environmental scarcity has pushed the research community to adopt new methods for the systematic management of clean water, as traditional techniques are not sufficient to remove pollutants such as fragments of organic dye waste in rivers.^7–11^ Photo-removal can represent the best, simplest, and most sustainable route for achieving the oxidation process using sunlight.^12–14^ Semiconductor-assisted photocatalysis is based on the fact that radicals and superoxides can be produced by photo-irradiation of the semiconducting materials, which in turn can oxidize and remove organic pollutants. The interaction of the radicals and superoxides with the organic materials is the key parameter of the degradation of hazardous materials into environmentally acceptable species.^15,16^ Metal oxides such as ZnO, SnO_2_, and TiO_2_, among others, have been studied intensively as promising photocatalysts, as they have many merits such as availability, photo- and thermal-stability, eco-friendly properties, excellent photocatalytic performance, and cost-effectiveness. Unfortunately, metal oxides suffer from two significant drawbacks that limit their use in photocatalytic applications; namely, they have a considerable bandgap energy (>3 eV) and rapid rates of electron–hole recombination, which reduce their photocatalytic performance. These drawbacks result in their exhibiting photo-activity only under UV irradiation, which represents a small percentage of the solar spectrum. Several strategies have been adopted to overcome these liabilities, such as metal doping, non-metal doping, co-doping using narrow-bandgap semiconductors or other metal oxides, dye sensitization, and formation of composites using 2D materials such as graphene and hexagonal boron nitride.^17–20^ The main challenge in this context is selecting a material with high semiconducting performance, high stability, recyclability, and biocompatibility. ZnS is an essential II–V group semiconductor that has intrigued scholars around the world owing to its numerous morphologies at the nanoscale, superior mechanical and physical properties, non-toxicity, and excellent photocatalytic properties. These properties have made ZnS a platform for a plethora of applications, for instance, light-emitting diodes (LEDs), wastewater treatment, hydrogen production, sensors, and biodevices.^21–23^ In addition to this, ZnS is an n-type semiconductor, water-insoluble, inexpensive, and has intrinsic transport properties (reduction of recombination and the resulting scattering of the carriers). There are two crystalline structures for ZnS, cubic and hexagonal (wurtzite), which have corresponding bandgap energies of 3.72 and 3.77 eV, respectively. Thus, the activity of ZnS has been limited to UV irradiation. The availability of ZnS has been a prime property of its utilization; ZnS can be prepared by several methods, such as supercritical-condition-based, sol–gel, electrochemical deposition, chemical vapor deposition, and co-precipitation methods.^24–26^ However, the template-assisted method has been found to be a facile, efficient, and economic strategy for the synthesis of nanomaterials, as it enables customization and tailoring of nanoparticles to preferred sizes, shapes, and dimensions.^27–29^ Seeding a photocatalyst over a chemical support is a promising way to optimize its photocatalytic activity by improving the dispersion of the nanoparticles over the support. This leads to the stabilization of the active sites and limits electron–hole recombination.^30,31^ The geometry of the photocatalyst depends principally on the type of support and the mode of preparation. Thus, the photocatalytic activity of ZnS nanoparticles can be enhanced and optimized in the UV region by the incorporation of ZnS nanoparticles on a high-surface-area support such as silica (SiO_2_). Therefore, microporous silica (SiO_2_) is considered to be an eco-friendly and economic support for the dispersion of ZnS photocatalysts, as it supplies the 3D spaces required to incorporate the nanoparticles and increase the specific surface area.^3,19,32–34^ Herein, ZnS was prepared using a wet precipitation method in the presence of CTAB, which was used as a pore-directing agent, together with *in situ* impregnation on silica nanoparticles.^35–38^ The photocatalytic activity of the fabricated ZnS/SiO_2_ nanocomposite was investigated through the photodegradation of the dyes methylene blue and eosin as pollutant models under UV irradiation, and complete degradation of both dyes was observed within 2 h. This can be ascribed to the change in the bandgap energy of ZnS resulting from its incorporation on the silica matrix. Chemical oxygen demand (COD) measurements confirmed the complete degradation of the organic pollutants into eco-friendly species, and the mechanism of pollutant degradation in the current setup has been investigated using a scavenger study. Density functional theory (DFT) calculations were employed to examine the optical features of the proposed photocatalyst (ZnS/SiO_2_), and their results were in agreement with the experimentally measured data for the prepared nanocomposite. Different proportions of ZnS were loaded onto the silica surface to determine the optimum percentage of ZnS required to enhance the photoactivity. Several characterization techniques were used to characterize and confirm the prepared nanocomposite materials.

## 2. Experimental section

### 2.1. Materials and methods

#### 2.1.1. Materials

Tetraethyl orthosilicate (TEOS), cetyltrimethylammonium bromide (CTAB), zinc acetate, sodium sulfide, hydrochloric acid, absolute ethanol, methylene blue (MB), and eosin (ES) were bought from Sigma-Aldrich with a high purity of approximately 99.99%.

#### 2.1.2. Synthesis of SiO_2_ nanoparticles

**Fig. 1:**
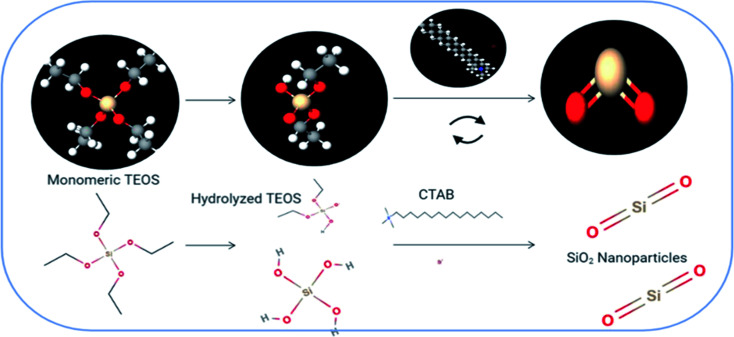
A schematic diagram representing the sol–gel synthesis of the silica network.

The silica nanoparticle network was synthesized *via* a sol–gel approach using tetraethyl orthosilicate (TEOS) as the silica precursor and CTAB as the pore directing agent. Ammonium hydroxide solution (10 mL) was incorporated as a co-precipitant. The formed sol was subjected to stirring for 2 h. Then, the sol was placed in a clean place for drying. The resulting gel was then dried overnight and washed many times using ethanol. The dried powder was then annealed at a temperature of 640 °C for 2 hours. A schematic diagram representing the sol–gel synthesis of the silica network is shown in Fig. 1.^2,39,40^

#### 2.1.3. Synthesis of ZnS blende nanoparticles

In the experimental preparation of ZnS, hydrogen sulfide (hydrochloric acid reduces sodium sulfide with the formation of hydrogen sulfide gas) was passed through a Zn(CH_3_COO)_2_·2H_2_O mixture until a white colloidal sol was observed. The above colloidal solution was stirred to obtain homogeneity, followed by filtration and washing with deionized water several times to get rid of any impurities.

#### 2.1.4. Synthesis of ZnS/SiO_2_ nanocomposites

A precise quantity of zinc acetate aqueous solution was seeded carefully into the prepared silica gel mixture with gentle stirring for two hours using ratios intended to result in the retention of 1, 15, 17, and 20 w/w% ZnS over a constant amount of silica gel. Moreover, hydrogen sulfide (H_2_S) gas was pushed through the above solution until a white sol was produced. The high sol was allowed to stand for a few days to make the powder drier. Filtration and washing with deionized water were repeated at various time intervals, and the precipitate was dried in an oven at 100 °C for a day to eliminate excess volatile compounds.

#### 2.1.5. Material characterization methodology

The powders of the various prepared photocatalysts were placed inside the stand of a P-Analytical X'PERT MPD diffractometer using Cu (Kα1/Kα2) X-rays; the diffraction angle spectrum was obtained from 5° to 80° with a constant step of 0.02°. In addition, volumetric instruments, as well as the Barret–Joyner–Halenda (BJH) approach, were used to detect the adsorption–desorption isotherms of N_2_ at specific low-temperature values of around 77 K under a low pressure of 10^−5^ torr. Furthermore, both field emission scanning electron microscopy (FESEM, JEOL 6340) and electron transmission microscopy (TEM, JEOL-JEM 1230) were used to characterize the prepared morphology of the composite powder and affirm the nanostructure of the synthesized materials. A JASCO spectrometer (V-570) was used to detect the optical properties and bandgap energies of the prepared samples as well as the visual features of the photocatalyst *via* ultraviolet-visible (UV-vis) spectra measurements. Additionally, theoretical DFT calculations were conducted to confirm the experimental data from the optical measurements. The photocatalytic activity of the prepared powders was determined by degrading MB and ES under a UV lamp with a monochromatic wavelength of 365 nm. The full details of the photocatalytic setup and procedure are provided in our previous study.^3^ Moreover, the terephthalic acid photoluminescence probing system [TA-PL] was applied for the detection of OH radicals, in which the basic TA solution was fixed into the reactor with a concentration of 5 × 10^−4^ M in a 2 × 10^−3^ M sodium hydroxide mixture. The system is based on the formation of fluorescent 2-hydroxyterephthalic acid [2-HTA] with irradiation contact time. The sample was collected every 20 min and studied using a spectrophotometer after careful separation. The fluorescence product was detected as a peak with a maximum at a wavelength of 423 nm. The chemical oxygen demand (COD) was characterized using a Bio block COD analyzer system.^41^

## 3. Results and discussion

### 3.1. Structural characterization of the materials

**Fig. 2:**
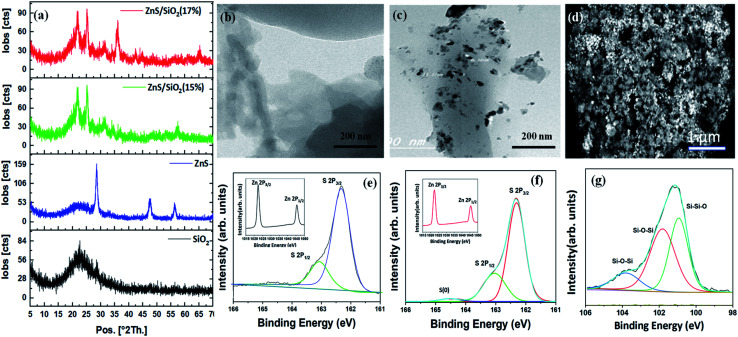
(a) XRD patterns of silica, the ZnS semiconductor, the ZnS/SiO_2_ photocatalyst (15%), and ZnS/SiO_2_ (17%). The data show the formation of amorphous silica with a broad peak at 2*θ* = 22°, as well as the presence of ZnS with the blende structure. A new peak corresponding to zinc silicate is also observed after increasing the concentration of ZnS to more than 15%. Zinc silicate formation limits the photocatalytic degradation of the organic dyes. (b and c) HRTEM images of silica and the ZnS/SiO_2_ nanocomposite. (d) A FESEM image of the as-prepared ZnS/SiO_2_ nanocomposite. A highly homogenous distribution of the particles over the whole silica matrix can be observed. (e and f) XPS analysis of Zn (inset) and S for the ZnS and ZnS/SiO_2_ (15%) samples and (g) the XPS spectrum of SiO_2_ in the ZnS/SiO_2_ (15%) sample.

The structures of the prepared materials were explored using XRD analysis. As can clearly be seen in Fig. 2(a), the XRD pattern of the prepared silica shows a broad XRD peak with an equivalent Bragg angle of 2*θ* = 22°, revealing the amorphous structure of this material. The smoothness and absence of other elements affirm that the repeated washings with DI water were very efficient in eliminating impurities that settled inside the pores of the gel network. It can also be seen that the XRD pattern of ZnS has three well-fitted diffraction peaks appearing at three different Bragg angles of 2*θ* = 28.51°, 47.62° and 56.4°, which were attributed to the (111), (220), and (311) planes of the cubic zinc sulfide blende structure (Fig. 2(a)). The crystalline planes matched very well with the face-centered cubic structure of the ZnS blende crystal structure described in ICDD PDF 65-1691, with a lattice parameter of 5.1 Å. In addition, as shown in Fig. 2(a), a ZnS/SiO_2_ photocatalyst XRD pattern was observed for different ratios of ZnS (15 and 17%); a broad silica peak at 2*θ* = 22° and the three other peaks of ZnS with varying intensities depending on the ZnS content of the sample (1–15%) were clearly observed in all the samples, which was highly consistent with the performance results and other features. A new peak corresponding to orthorhombic zinc silicate can also be seen at 2*θ* = 36° for samples containing more than 15% ZnS, as shown in Fig. 2(a). It is essential to mention that the formation of Zn_2_SiO_4_ requires reaction between at least two moles of ZnS and one mole of silica, which explains why the zinc silicate structures are created when the amount of ZnS is increased. The formation of zinc silicates was the key factor for the lower dye degradation rate associated with samples with more than 15% ZnS. High resolution-TEM (HRTEM) images show the nanoscale structure of ZnS/SiO_2_. Careful study of Fig. 2(b) shows the presence of highly porous silica, consistent with the XRD data. Fig. 2(c) illustrates that the spherical ZnS particles with a size of less than 30 nm are dispersed with a satisfactory degree of homogeneity throughout the silica network matrix. The degree of homogeneity can be confirmed from the FESEM image, in which we can see clearly the spherical ZnS particles arranged well throughout the silica gel clouds, as shown in Fig. 2(d). The variation in the chemical binding status of the ZnS nanoparticles was examined through XPS analyses. Fig. 2(e and f) present the XPS surface narrow scan Zn and S spectra of the prepared ZnS and ZnS/SiO_2_ (15%) samples. The narrow-scan XPS shows peaks at binding energies (BE) of 1044 and 1021 eV with a doublet separation of almost 23.1 eV, which are attributed to the Zn 2p_1/2_ and Zn 2p_3/2_ peaks, respectively, for Zn^2+^. Furthermore, the asymmetric S 2p peak in Fig. 2(e) was deconvoluted into two subpeaks consistent with S 2p_3/2_ and S 2p_1/2_ at 162.1 and 163.1 eV, respectively. An extra S peak corresponding to an S oxidation state of zero appeared in the ZnS/SiO_2_ (15%) heterostructure. Additionally, the peak with a binding energy of 161.1 eV arose from S^2−^ in the ZnS structure, and the subpeak at approximately 163.1 eV was attributed to the S–S species surface defects in the ZnS sample. In Fig. 2(g), we can clearly see the XPS spectrum of the silica gel in the composite; the peaks at 102.5 eV and 103.7 eV are associated with Si–O–Si. The peak at 100.6 eV could correspond to Si–Si–O, which can be clearly observed.

### 3.2. Surface area (*S*_BET_) characterization

**Fig. 3:**
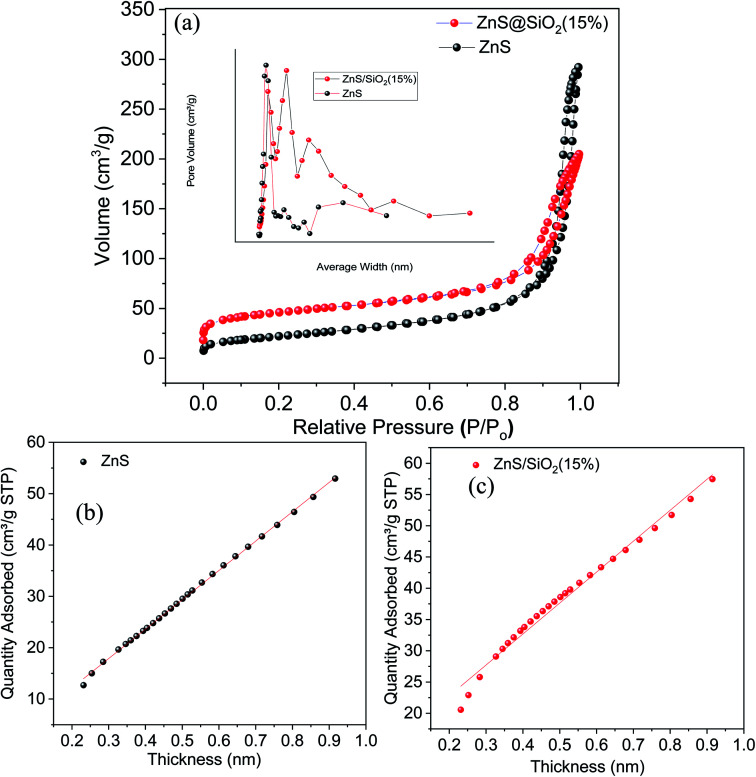
(a) N_2_ adsorption–desorption isotherms and pore size distributions and survey data, and (b & c) *V*–*t* (thickness) plots for the ZnS photocatalyst and ZnS/SiO_2_ (15%) photocatalyst. The results show an increased surface area of around 150 m^2^ g^−1^ for ZnS/SiO_2_, which is sufficient for photocatalysis-based reactions.

As mentioned above the N_2_ isotherms and pore size distribution were measured at a low temperature close to 77 K, and the isotherms for the silica, ZnS, and ZnS/SiO_2_ (15%) samples can be seen in Fig. 3(a). The adsorption isotherm of silica was of type IV according to the IUPAC classification system, exhibiting an H1 hysteresis direction that closes at *P*/*P*_o_ = 0.452. This category of hysteresis is considered to involve two different branches that appear vertically very close to one another over an appreciable range of gas uptake, and arises from the presence of cylindrical pores that are open at both ends as shown in Fig. S1 in the ESI.† Furthermore, the adsorption isotherms of the ZnS and ZnS/SiO_2_ (15%) materials are classified as type II with a very narrow hysteresis loop (Fig. 3(a)). Additionally, the specific surface areas (*S*_BET_) of the silica, ZnS, and ZnS/SiO_2_ (15%) materials are 805.9, 153.2, and 92 m^2^ g^−1^, respectively, as measured by using the BET equation for these materials. From there, the pore volume was determined at the saturation pressure and was expressed as a liquid volume of 0.817 and 0.438 cm^3^ g^−1^ for ZnS and ZnS/SiO_2_ (15%), respectively. The incorporation of the ZnS semiconducting materials inside the silica matrix was found to affect the nitrogen sorption features of silica, changing the isotherm from a type IV isotherm associated with mesoporous solids to type II, as mentioned above. This change in texture translates into a reduction in the surface area and the total pore volume. The noteworthy reduction in the texture characteristics of silica indicates the high potential connection between the ZnS and SiO_2_ materials, as the primary target for ZnS particles is the hydroxide groups that diffuse over the surface of silica and inside the pore network, decreasing the available surface area and narrowing the pore structure. In addition, the variance in the constant *C* affirms these results, as its value varies from 59.3 to 33.5 for SiO_2_ and ZnS/SiO_2_ (15%), respectively. This reveals that the decrease in surface polarity is related to the integration of ZnS nanoparticles into the matrix. Based on this isotherm, the surface area per gram of these photocatalysts follows the order ZnS (92 m^2^ g^−1^) < ZnS/SiO_2_ (154 m^2^ g^−1^) < SiO_2_ (800 m^2^ g^−1^). Moreover, the BJH average pore sizes were calculated based on the average adsorption and desorption pore size values, with the average pore diameter following the order ZnS (9.39 nm) < ZnS/SiO_2_ (14 nm) < SiO_2_ (20 nm).

Furthermore, the porosity study results of the volume absorbed *versus* the thickness for the ZnS photocatalyst and ZnS/SiO_2_ (15%) photocatalyst are illustrated in Fig. 3(b and c). The *V*–*t* curves for pure silica and ZnS show a downward deviation for pure silica, indicating the predominant existence of micropores. The insertion of ZnS into the silica matrix is accompanied by the disappearance of these micropores due to deposition of ZnS photocatalyst over the silica and a change in sample texture from microporous to mesoporous. The surface factors show a reduction in the surface area and pore volume values and an increase in the pore thickness values, which reflect the deposition of the ZnS material over the walls of the silica micropores with a high potential affinity to fill the microporous texture. In addition, the pore sizes of the silica and silica-based composites were evaluated using the Barret, Joyner, and Halenda (BJH) method, as illustrated in the inset of Fig. 3(a). The existence of a narrow peak for pure silica indicates that all the pores in the solid sample have almost the same size. However, the insertion of ZnS nanoparticles into the silica matrix leads to the development of many peaks, the first of which is centered at 20 Å and corresponds to supermicropores, while the broad peak centered at 28 Å indicates a wide spectrum of porosity.

### 3.3. Optical characteristics and DFT calculations

**Fig. 4:**
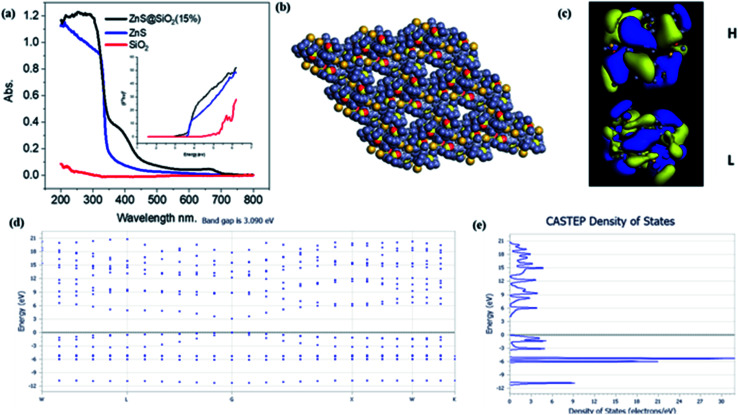
(a) Diffuse reflectance spectra (DRS) of SiO_2_, ZnS, and ZnS/SiO_2_ (15%); the inset shows the Tauc plots. Representations of the DFT calculations of the prepared composite: (b) the ZnS/SiO_2_ material unit cell structure, (c) the HOMO and LUMO structures, (d) the band structure and (e) the density of states. The theoretically calculated band gap energy value is very close to the experimental value.

The diffuse reflectance spectra (DRS) of silica, ZnS and the ZnS/SiO_2_ (15%) nanocomposite photocatalyst are illustrated in Fig. 4(a). The obtained results reflect the presence of a sharp band step in the UV-Vis portion at 200–300 nm for silica and at 350–400 nm for both ZnS and ZnS/SiO_2_ (15%), corresponding to photoexcitation from the lower valence band to the higher conduction one. By scrutinizing the results, one can perceive that the ZnS/SiO_2_ (15%) sample exhibits the same shift toward the visible-light regions as the ZnS semiconducting material, which confirms that the semiconductive property of the ZnS remains the same even after distribution over the silica network. According to the bandgap structure measurements from the Tauc diagram and the equation (*αhv*)^2^ = *A*(*hv* − *E*_g_)_*n*_, where *α* is the absorption factor, *A* is a constant, and *n* = 2 for direct transition and *n* = 1/2 for indirect change, the bandgap values (*E*_g_) are 5.6, 3.6 and 3.2 eV for the pure silica, ZnS, and ZnS/SiO_2_ (15%), respectively, as shown in the inset of Fig. 4(a). This result removes any idea about the effect of silica on the bandgap energy of ZnS. The rationale behind the approach was to increase the surface area without affecting the bandgap energy. In addition, Fig. 4(b–e) presents the theoretical insights into the band sites. DFT calculations were carried out using the Cambridge sequential total energy package (CASTEP) in Materials Studio. The generalized gradient approximation (GGA) of the Perdew–Burke–Ernzerhof (PBE) functional was used to describe the exchange–correlation interactions. The DFT-D3 method was adopted to consider the van der Waals (vdW) interactions. For geometric optimization, the convergence thresholds for energy and force were set to 10^−5^ eV per atom and 0.02 eV Å^−1^, respectively. A cutoff energy of 550 eV was chosen for a plane wave basis. A Monkhorst mesh of 3 × 3 × 1 *k*-points sampled the Brillouin zone. The ZnS/SiO_2_ was modeled as a ZnS cluster supported on single-layered SiO_2_ (S, 4 × 4 × 1 supercell) with monovacancy. Representations of the DFT calculations of the prepared composite can be found in the material unit cell structure in Fig. 4(b), the HOMO and LUMO structures Fig. 4(c), band structure in Fig. 4(d) and density of states in Fig. 4(e) for the ZnS/SiO_2_ nanocomposite material. The ZnS/SiO_2_ (15%) system was found to have a bandgap energy of approximately 3.02 eV, which is close to the experimentally calculated value. This confirms that silica has no effect on the band structure of the prepared composite. According to these theoretical DFT calculations, the band gap energy of ZnS after adding silica is 3.09 eV, which is very close to the experimental value. In most cases, the separation and recombination of electron–hole pairs are in continuous competition. In addition, the photodegradation process for the oxidation of organic pollutant materials is generally improved through greater separation between electrons and holes to increase their lifetime. According to the photoluminescence (PL) spectra of ZnS and ZnS/SiO_2_ (15%), an intense prevailing peak can be observed at 440 nm, which originates from the various defects in the ZnS crystalline structure (Fig. S2, ESI†). After the incorporation of ZnS in the silica gel matrix, remarkable quenching of the ZnS peak is observed, reflecting the strong interaction between the silica and zinc sulfide material. Fig. S3† shows the experimental determination of the HOMO and LUMO values of the as-prepared ZnS/SiO_2_ using electrochemistry, in which the valence band was found to be located at 2.1 eV and the conduction band at −1.2 eV.

### 3.4. Photocatalytic degradation of organic pollutants (using MB and ES as models)

#### 3.4.1. Kinetics of pollutant degradation

**Fig. 5:**
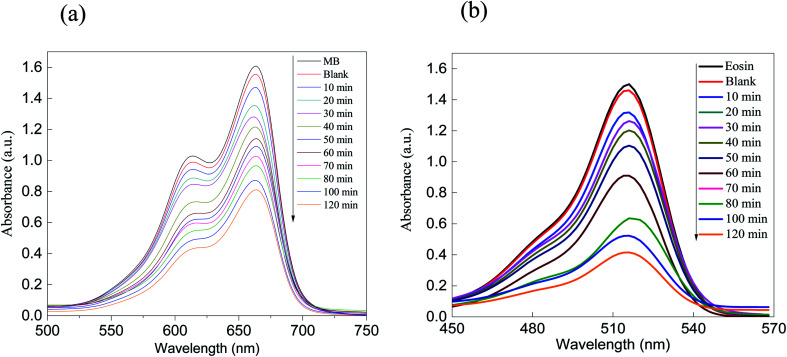
Absorption spectra of the removal of the dyes (a) MB and (b) eosin over ZnS. As shown, the removal performance rate is greater than 50% after 120 minutes of irradiation.

**Fig. 6:**
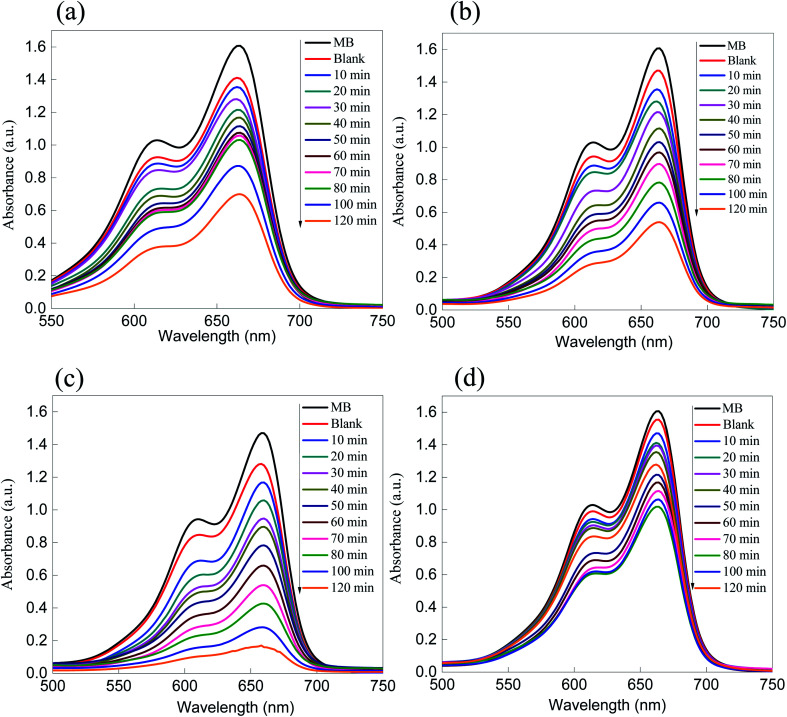
Absorption (A) spectra of the photo-removal of MB over (a) ZnS/silica (5%), (b) ZnS/silica (10%), (c) ZnS/silica (15%), and (d) ZnS/silica (17%), showing a continuous increase in the removal rate from 5–15%, with inhibition at higher ratios due to the blocking of active sites.

**Fig. 7:**
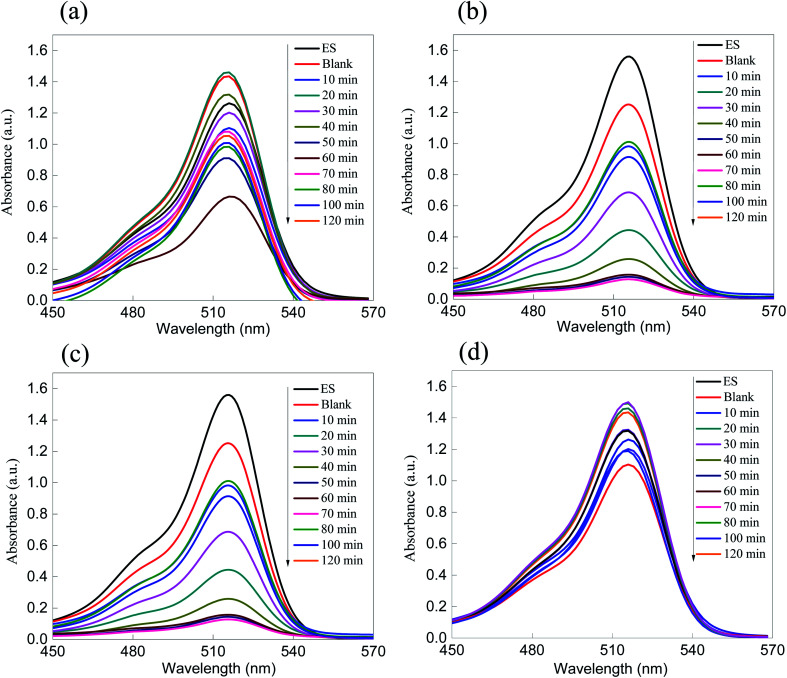
Absorption spectra of the photo-removal of ES over (a) ZnS/silica (5%), (b) ZnS/silica (10%), (c) ZnS/silica (15%), and (d) ZnS/silica (17%), showing a continuous increase in the removal rate from 5–15%, with inhibition at higher ratios resulting from the blocking of the active sites.

The photocatalytic activity of the ZnS semiconducting material as well as that of the ZnS/SiO_2_ photo-nanomaterial was evaluated using the removal rate of MB as a cationic model (+) and eosin (ES) as an anionic model (−). The amounts of the organic dye pollutants were set to be consistent with the industrial context, approximately at the microscale, and they were constantly stirred in the presence of the photocatalyst. The absorption spectra of both organic dye models were monitored using UV-vis spectroscopy *via* two characteristic peaks at two different wavelengths (*λ*_max_ = 664 nm and *λ*_max_ = 518 nm for the MB and ES organic dye models, respectively). Fig. 5(a and b) present the absorption (*A*) spectra of the photocatalytic removal of MB and ES dyes over ZnS as a photocatalyst. The photocatalytic removal rate of both dyes over the ZnS photocatalyst showed suitable values that reached approximately 50% and 55% for MB and ES, respectively. The challenge here is to increase the surface area without affecting the optical features of the semiconducting material. In addition, the as-prepared ZnS/SiO_2_ photocatalysts with different weight ratios of ZnS (5, 10, 15, and 17%) were tested for the photodegradation of the different organic dye model pollutants (MB and eosin), as shown in Fig. 6 and 7. It is interesting to note that the removal rate improved with increasing ZnS quantity up to 15 wt%, followed by a noticeable reduction in the reactivity for the samples with 17% and 20% of embedded ZnS. The results for both organic dyes showed that the removal rate reached approximately 98% when the dyes were treated with ZnS/SiO_2_ (15%), but less than 50% when they were treated with the materials with ZnS ratios of 17% and 20%, which was attributed to blocking of the active sites above 15%.

**Fig. 8:**
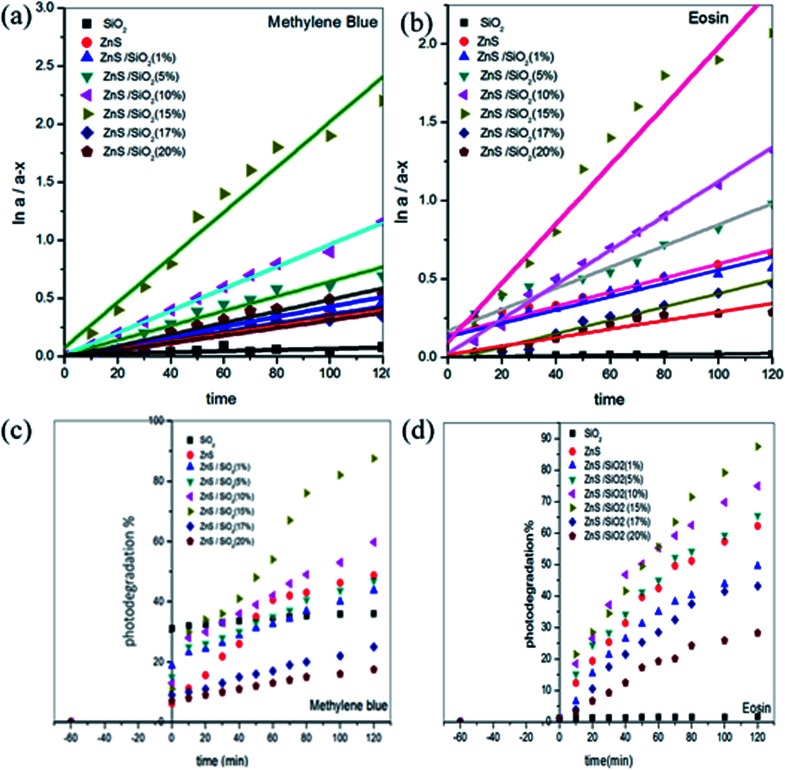
(a and b) Pseudo-first-order kinetics of the removal of the organic dyes (a) MB and (b) ES over SiO_2_, ZnS, and ZnS/silica prepared with different ratios. (c and d) Variations of the photo-removal rates of the dyes (c) MB and (d) ES.

The chemical kinetics of the photodegradation rates obey first-order reaction kinetics, as illustrated in Fig. 8(a and b), where the rate constant is expressed using the following formula:*C* = *C*_o_ exp(−*kt*) or ln *C*/*C*_0_ = −*kt*,*C*_o_ is the initial molar concentration of the dye (mol L^−1^), *C* is the molar concentration of the dye at time *t* (mol L^−1^), *t* is the irradiation time (minutes), and *k* is the rate constant. Plotting ln *C*_o_/*C versus* contact time provides the rate constant, as shown in Fig. 8(a and b). In addition, the rate constants in the case of MB were calculated to be 5.04 × 10^−4^, 0.00335, 0.00362, 0.00598, 0.01282, 0.01941, 0.00291 and 0.00158 min^−1^ and in the case of ES, 1.79 × 10^−4^, 0.00452, 0.00425, 0.00677, 0.011, 0.0188, 0.00428 and 0.00274 min^−1^, for pure SiO_2_, ZnS, ZnS/SiO_2_ (1%), ZnS/SiO_2_ (5%), ZnS/SiO_2_ (10%), ZnS/SiO_2_ (15%), ZnS/SiO_2_ (17%) and ZnS/SiO_2_ (20%), respectively, as shown in Fig. 8(c and d).

Subsequently, from the photocatalytic performance, it can be observed that more than 90% of the organic dye was completely removed after approximately 2 hours, which is very efficient compared to reports in the literature, as shown in Table 1.

**Table 1:** Various reported photocatalytic studies for the removal of organic dyes

	Photocatalyst	Pollutant model	Degradation%	Contact time	Reference
1	ZnO/CuO	Textile dye	100%	120 min	42
2	Carbon nanotubes and fullerene	Wastewater	—	—	43
3	V_2_O_5_/ZnO	Organic pollutants	90%	120 min	44
4	ZnO	Organic pollutants	95%	180 min	45
5	ZnO/γ-Mn_2_O_3_	Textile effluent	100%	8 hours	46
6	ZnS:Cu	Basic dye Auramine-O	100%	—	47
7	Fe_3_O_4_	Methylene blue and Safranin-O	—	—	48
8	Synergetic effect of adsorption	Wastewater	—	—	49
9	PMMA composites	Phenols	—	—	50
12	ZnO	Organic dyes	100%	120 min	51
13	C_3_N_4_@nickel–aluminum	Organic dyes	100%	—	52
14	ZnO microcrystals	Organic dyes	85%	—	53
15	Nitride/metal–organic frameworks (MOFs)	Organic dyes	100%	—	54
16	Ag-doped TiO_2_	Azo dyes	95%	120 min	55
17	Ni-doped ZnS	Organic dyes		—	56
18	ZnS	—		—	57
19	CdS–ZnS/TiO_2_ combined	Organic dyes	90%	120 min	58
20	ZnS:Ni_2_^+^	Organic dyes	95%	120 min	59
21	ZnS/SiO_2_	Organic dyes	97%	120 min	The current work

#### 3.4.2. Detection of hydroxyl radicals and effect of radical scavengers

The terephthalic acid photoluminescence [PL] probing system was used to detect the presence of OH radicals and their role in removing the organic dye waste from the water. It is well known that terephthalic acid is considered to be a non-fluorescent material, but it has a valuable function as it can bind with OH to give 2-hydroxyterephthalic acid, which exhibits a definite fluorescence peak.^3,19^ Therefore, we measured the PL spectra of the organic dye mixture after UV exposure for different time intervals using terephthalic acid.

**Fig. 9:**
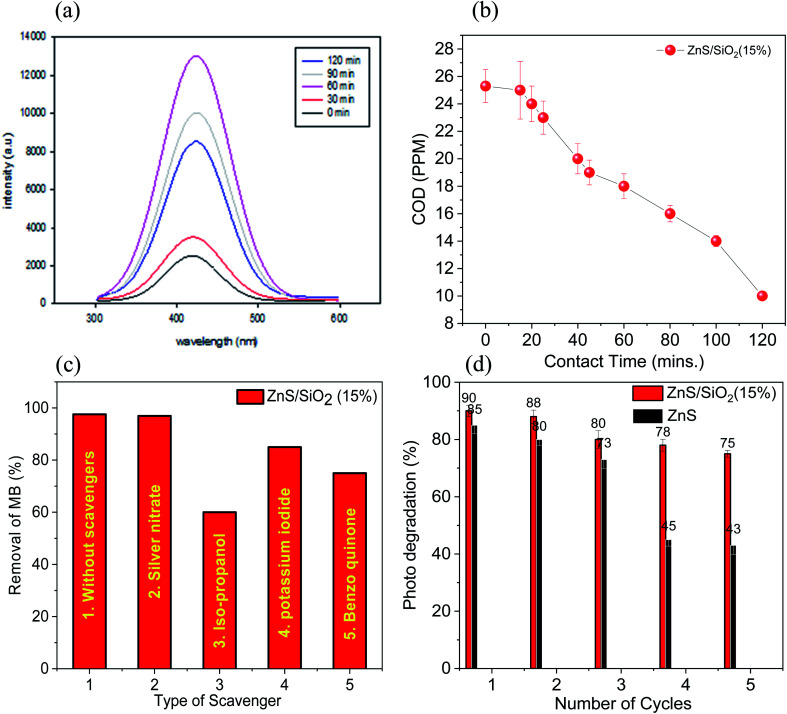
(a) PL spectral differences observed during the irradiation of ZnS/SiO_2_ (15%) in 5 × 10^−4^ M solution (315 nm), (b) the COD reduction rate, (c) the effects of various scavengers in the presence of ZnS/SiO_2_ (15%), and (d) the regeneration of ZnS/SiO_2_ (15%) nanoparticles over five successive cycles.

Fig. 9(a) demonstrates the prevailing peak at 423 nm, which increased in intensity with increasing irradiation time over the ZnS/SiO_2_ (15%) sample, demonstrating the high level of OH radical production. The chemical oxygen demand (COD) test allows us to check the organic content in water as a function of the irradiation time. It is also considered to be an excellent indicator to calculate the organic pollution *via* the total quantity of oxygen required for its oxidation to CO_2_ and H_2_O. The chemical oxygen demand test was performed on the dye solution before, during and after the photocatalytic removal process. The COD of the solution of the organic dye pollutant MB was found to decrease from 25.6 ppm to 14.3 ppm after 90 min and 8.2 ppm after 120 min of irradiation with UV light over ZnS/SiO_2_ (15%). The COD results are consistent and fit very well with the removal rate results, indicating the complete photo-based degradation of MB into inorganic species, as shown in Fig. 9(b). Total organic carbon (TOC) is a measure of the total amount of carbon in organic compounds in pure water and aqueous systems. The TOC was measured for the MB mixture over ZnS/SiO_2_ (15%), and we found that the TOC at 0 contact time was 24.6 ppm, and decreased to 16.5 ppm after 30 minutes and to 7.8 ppm after 1 hour. This confirms the organic content removal with increasing contact time.

Useful species responsible for the removal rate, such as h^+^, OH species, e^−^, and superoxide, were measured using KI, isopropanol, Ag nitrate, and benzoquinone in the presence of the ZnS@SiO_2_ (15%) nanocomposite photocatalyst. The reaction conditions were adjusted as follows: 0.1 g of the photocatalyst was added to 100 mL of the dye stock solution (2 × 10^−5^ mol L^−1^), and irradiated for 2 hours in the presence of the radical scavengers with a concentration of 10^−3^ mol L^−1^. The results established that the removal rate of the MB organic dye model was reduced from 97.6% to 85, 75, 60, and 95% after the addition of KI, benzoquinone, isopropanol, and silver nitrate scavengers, as shown in Fig. 9(c). The results indicate that hydroxide radicals are more predominantly responsible for the photocatalytic degradation of the organic dye pollutant MB over the ZnS/SiO_2_ (15%) nanocomposite photocatalyst. We measured the point of zero charge (PZC) to supplement the discussion; from zeta potential measurements, it was noted that the point of zero charge has an essential effect on the photocatalytic performance. The PZC values were 4.2 and 5.6 for ZnS and ZnS/SiO_2_ (15%), respectively. The mechanism of the photocatalytic reaction showed the effect of the reaction medium on the photo-removal reactivity for both MB and eosin over ZnS/SiO_2_. However, at low pH values (below 5.9), the surface is positively charged, which leads to repulsion with cationic dyes such as MB and intense interaction with anionic dyes such as ES. Thus, we can see more degradation for ES at lower pH. By increasing the pH to above 5.9, more OH groups accumulated on the surface, which becomes more negatively charged after some time. This will increase the degradation percentage of MB dye relative to that of ES.

#### 3.4.3. ZnS/SiO_2_ (15%) reusability

Photocatalyst recycling is a critical parameter for commercial use; the photocatalyst must retain its stability and activity after multiple cycles. The reuse procedure for removing MB dye was performed to determine the stability of the ZnS/SiO_2_ (15%) composite after five successive cycles. The obtained results demonstrated that the prepared ZnS/SiO_2_ (15%) composite retains 82% of its reactivity after five cycles, revealing its high stability compared to the ZnS material, as shown in Fig. 9(d).

## 4. Conclusions

ZnS loaded on SiO_2_ is considered to be a promising heterostructure for the removal of MB and ES dyes, which act as organic pollutant models. The photocatalytic procedure is typically conducted by exposing the photocatalyst surface to UV irradiation, which generates reactive radicals. However, electron–hole recombination and a wide bandgap energy are the main challenges that reduce the photo-reactivity during the removal of organic pollutants. Herein, a promising route to augment the photocatalytic reactivity of ZnS *via* its homogenous distribution over a supporting material to increase the photo-reactivity and reusability is reported. This kind of support plays a decisive role in the photocatalytic degradation of the organic dye. The photocatalytic removal kinetics show that the MB and ES dyes are almost completely removed after approximately 120 minutes of irradiation treatment over the ZnS/SiO_2_ (15%) photocatalyst. Furthermore, COD measurements also confirm the organic content removal *via* calculating the oxygen uptake. To better understand the mechanism of action, radical scavenger studies were carried out to determine the active species in the process. It was found that OH radicals and superoxide were the predominant species in the removal process. Additionally, a reusability study of the catalyst affirmed that it can be reused with an efficiency of more than 50% after five cycles. Overall, ZnS/SiO_2_ (15%) proved to be a promising catalyst for water pollutant photo-treatment.

## Conflicts of interest

There are no conflicts to declare.

## Supplementary Material

RA-011-D1RA02284A-s001
