# Expression of Concern: The controlled synthesis and DFT investigation of novel (0D)–(3D) ZnS/SiO_2_ heterostructures for photocatalytic applications

**DOI:** 10.1039/d3ra90114a

**Published:** 2023-11-08

**Authors:** Mohamed F. Sanad, Ahmed Esmail Shalan, M. A. Ahmed, M. F. Abdel Messih

**Affiliations:** a Chemistry Department, Faculty of Science, Ain Shams University Egypt mfsanad@miners.utep.edu; b Department of Chemistry, Department of Environmental Sciences and Engineering, University of Texas at El Paso 500 West University Avenue El Paso Texas 79968 USA; c Central Metallurgical Research and Development Institute (CMRDI) P.O. Box 87 Helwan Cairo 11422 Egypt a.shalan133@gmail.com ahmed.shalan@bcmaterials.net; d BCMaterials, Basque Center for Materials, Applications and Nanostructures, Martina Casiano, UPV/EHU Science Park Barrio Sarriena s/n Leioa 48940 Spain

## Abstract

Expression of Concern for ‘The controlled synthesis and DFT investigation of novel (0D)–(3D) ZnS/SiO_2_ heterostructures for photocatalytic applications’ by Mohamed F. Sanad *et al.*, *RSC Adv.*, 2021, **11**, 22352–22364, https://doi.org/10.1039/D1RA02284A.

The Royal Society of Chemistry is publishing this expression of concern in order to alert readers that concerns have been raised regarding the reliability of the XRD data in Fig. 2a, the absorption spectra in Fig. 6, 7b and c, and the kinetics data in Fig. 8a and b. An investigation is underway, and an Expression of Concern will continue to be associated with the article until a final outcome is reached.

Laura Fisher

2^nd^ November 2023

Executive Editor, *RSC Advances*

## Supplementary Material

