# Supplementary material for: The controlled synthesis and DFT investigation of novel (0D)–(3D) ZnS/SiO2 heterostructures for photocatalytic applications
Source: RSC Adv. 2021 Jun 24;11(36):22352–64. doi: 10.1039/d1ra02284a (PMC9034197; doi:10.1039/d1ra02284a)
Supplement: RA-011-D1RA02284A-s001 [file RA-011-D1RA02284A-s001.pdf]

Electronic Supplementary Material (ESI) for RSC Advances.
This journal is © The Royal Society of Chemistry 2021

Supplementary Information

Controlled Synthesis and DFT Insights Investigation of Novel 0(D)-(3D) ZnS/SiO_2 Heterostructure for Photocatalytic Applications

Mohamed F. Sanad[1,2,,+], Ahmed Esmail Shalan[3,4,*,+], M. A. Ahmed[1], M. F. Abdel Messih[1]*

[1] Chemistry department, Faculty of Science, Ain Shams University, Egypt.

[2] Department of Chemistry and Department of Environmental Sciences and Engineering, University of Texas at El Paso, 500 West University Avenue, El Paso, Texas 79968, United States.

[3] Central Metallurgical Research and Development Institute (CMRDI), P.O. Box 87, Helwan, Cairo, 11422, Egypt.

[4] BCMaterials, Basque Center for Materials, Applications and Nanostructures, Martina Casiano, UPV/EHU Science Park, Barrio Sarriena s/n, Leioa 48940, Spain

[+] These authors contributed equally to the work.

[*]Corresponding authors

A. E. Shalan; E-mail address: a.shalan133@gmail.com; ahmrd.shalan@bcmaterials.net

M. F. Sanad; E-mail address: mfsanad@miners.utep.edu

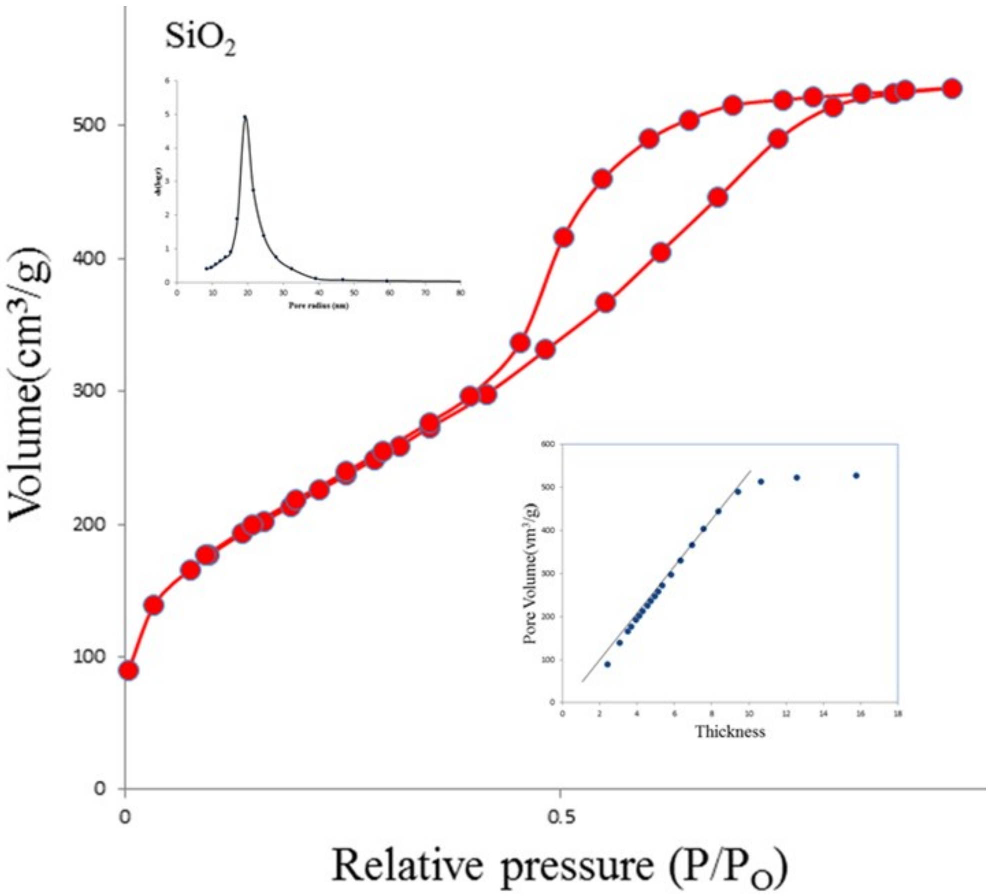

Figure S1: N_2-adsorption-desorption isotherm, V-t (thickness) plot and pore size distribution and survey for SiO_2. Results dedicates high surface area silica with type 4 isotherm with high surface area close to 800 m^2/g.

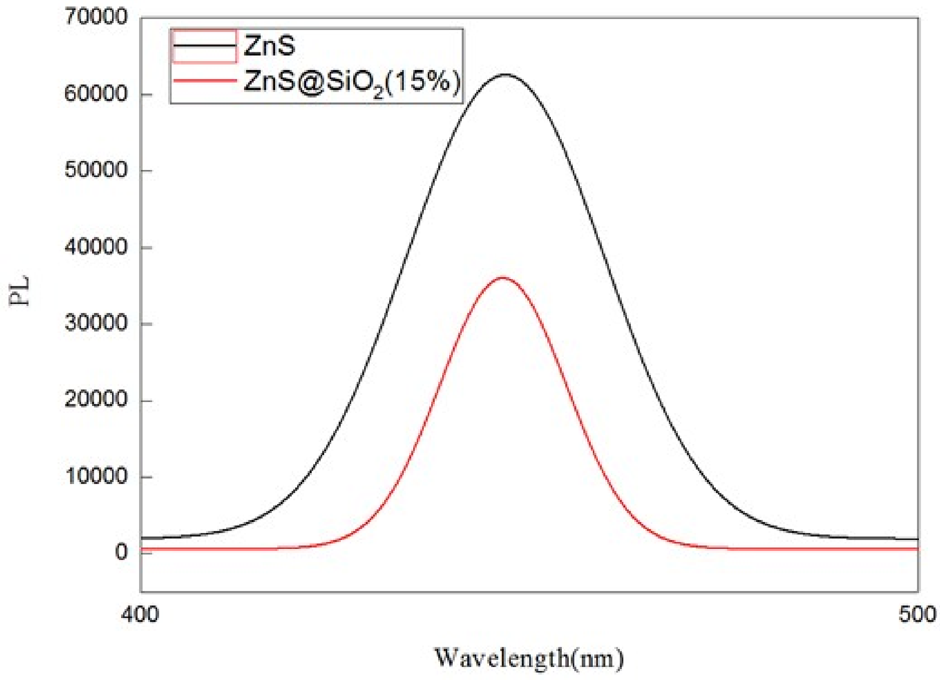

Figure S2: Photoluminescence (PL) spectra of ZnS and ZnS/SiO_2 (15%).

Pl reflects the defect appeared in the material lattice. Usually there is a competition between separation and re-combination of electron-hole pairs. In addition, the photo-degradation process is generally improved through the higher separation between electron and hole to increase its lifetime in the oxidation of organic pollutant materials. According to the Figure, the presence of a prevailing peak at 440 nm can be observed intensively, which come from the different defects in the ZnS crystalline structure. After incorporation of ZnS in silica gel matrix, a remarkable quenching of the ZnS peak can be observed reflecting the strong interaction among silica and zinc sulfide material.

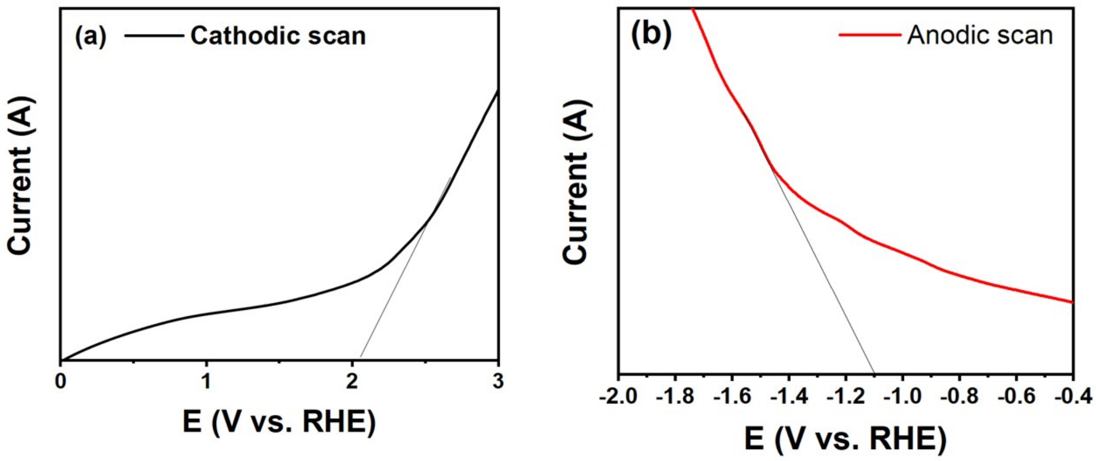

Figure S3: Anodic and cathodic LSV scan of ZnS/SiO_2 (15%).
